# A systematic review of the impact of the COVID-19 pandemic on the mental health of adolescents and young people with disabilities aged 15–29 years

**DOI:** 10.1186/s12889-023-16260-z

**Published:** 2023-07-19

**Authors:** Xing Yu Leung, Anne Marie Kavanagh, Que Tien Quang, Marissa Shields, Zoe Aitken

**Affiliations:** Melbourne School of Population and Global Health, The University of Melbourne, Parkville, Australia

**Keywords:** COVID-19, Disability, Adolescents, Young adults, Mental health

## Abstract

**Background:**

The COVID-19 pandemic has exacerbated the psychological burden on young people around the world and may have disproportionately large impacts for young people with disabilities. This review aims to systematically review the quantitative evidence on the impact of the COVID-19 pandemic on the mental health of young people with disabilities and evaluate the quality of included studies.

**Methods:**

A systematic search was conducted using 5 electronic databases. The quality of the studies was assessed using the SIGN risk of bias assessment tool. A narrative synthesis was performed to synthesize the results of included studies.

**Results:**

The initial search yielded 1935 studies, of which two met the eligibility criteria, one longitudinal study and one cross-sectional study, both assessed to be of low quality. In the cross-sectional study, young people with intellectual and developmental disabilities self-reported an increase in mental health symptoms. The longitudinal study found no evidence of a change in mental health symptoms from pre-pandemic to during the pandemic among young people with autism spectrum disorder, although these individuals reported negative impacts of the COVID-19 pandemic on their emotional or mental health.

**Conclusions:**

The findings of this review provide some weak evidence of a negative impact of the COVID-19 pandemic on the mental health of young people with disabilities. Importantly, the findings highlight the lack of research in this area. More research is needed to investigate the impact of the pandemic on the mental health of young disabled people, in order for governments to develop emergency preparedness plans to safeguard the well-being of this population.

**Supplementary Information:**

The online version contains supplementary material available at 10.1186/s12889-023-16260-z.

## Background

Coronavirus disease (COVID-19) is an infectious respiratory disease caused by the SARS-CoV-2 coronavirus, which was first detected in the city of Wuhan, Hubei Province of China, in December 2019 [1]. Due to its high morbidity and mortality, COVID-19 was declared a pandemic by the World Health Organization (WHO) on 11 March 2020 [2]. With the spread of COVID-19 and the rapid increase in confirmed cases worldwide, countries revised their public health strategies to prevent the transmission of the virus, including mask wearing, lockdowns and stay at home orders, and social distancing [3]. The rapid implementation of these preventative approaches has had a profound influence on people's lives. It has led to social isolation, increased risk of unemployment, financial stress, disruption to education, media information overload, and uncertainty and fear about the future, all of which are associated with poorer mental health [4, 5].

According to the WHO, mental health is defined as a state of well-being in which individuals are able to fulfil their potential, cope with regular stresses in life, learn and work effectively, and contribute to their community [6]. A recent review summarised the evidence relating to mental health problems during the COVID-19 pandemic, and found that the pandemic was associated with increasing symptoms of depression, anxiety, stress, irritability, panic, sleep problems, suicidal behaviours and post-traumatic stress disorder, especially for adolescents and young adults [7–9].

Adolescence is an important life stage, a time in which young people acquire capabilities and resources that underpin mental health across their lifespan [10]. Adolescents and young adults are particularly vulnerable to mental health problems and are likely to be at increased risk of detrimental mental health impacts of such preventative measures [11]. As such, it is important to understand the mental health impacts of the COVID-19 pandemic on young people and how the impact may vary for different subgroups of this population.

One group that may be particularly at risk of poor mental health problems are adolescents with disability. This review conceptualizes disability based on the biopsychosocial model of disability described in the WHO International Classification of Functioning, Disability and Health, where disabilities are defined as health conditions or impairments that hinder functioning as a result of the interaction between people’s health conditions and the society in which people live. In line with this conceptualization, disability is defined in terms of functioning rather than underlying health conditions [12, 13]. Currently, there are more than one billion people living with a disability globally, accounting for approximately 15% of the world's population, and one-third of them are young adolescents and young adults [13, 14].

Like people with disabilities in general, young people with disabilities are more likely to experience social and economic inequalities in relation to those without disabilities, and are at greater risk of poor mental health [12, 15]. Evidence suggests that their poorer mental health results, in part, from inequitable access to health services, socio-economic disadvantage (e.g., poverty, low education, higher rates of unemployment, stigma and discrimination), and lower levels of social support [16–22]. Firstly, young people with disabilities generally have higher healthcare needs compared to young people without disabilities [12, 15]. However, they experience barriers in accessing health services due to long waiting times, high costs, lack of communication and discrimination by health professionals, [12, 19] which have been exacerbated during the COVID-19 pandemic [22]. Secondly, socio-economic inequalities are also likely to have been exacerbated during the COVID-19 pandemic [14, 23–26]. Young people with disabilities have lower levels of education, [12, 24] due to barriers in the existing education system, inadequate resources to address the needs of young people with disabilities, and discrimination [12, 24]. Prolonged school closures during the pandemic and an increasing reliance on online learning may have presented an additional barrier for young people with disabilities [27]. For young people with disabilities in the labour market, increasing unemployment rates and decreased job security may have had a greater impact on young people with disabilities compared to people without disabilities, [28] with implications for poverty and financial stress [12, 28]. Thirdly, young people with disabilities are more likely to be socially isolated compared to those without disabilities, experiencing low levels of social support, less social contact with family and friends, and increased feelings of not being part of the community, which is likely to have been exacerbated during the COVID-19 pandemic [20–24].

As such, young people with disabilities may experience disproportionately large mental health impacts associated with the COVID-19 pandemic, further widening existing inequalities, because of the impacts of the pandemic on these known determinants of mental health including access to health services, socio-economic disadvantage, and poor social support [22–24]. A growing number of studies have been published examining the impact of the COVID-19 pandemic on the mental health of young people with disabilities [29–33]. Yet, no systematic review has been performed to summarise the evidence, which is necessary for providing a more comprehensive understanding of the mental health impact of COVID-19 on young people with disabilities. The review synthesizes the available evidence to identify research gaps and lays a foundation for improving future research work in this topic area. Therefore, this review aims to systematically review the published literature on the impact of the COVID-19 pandemic on the mental health of adolescents and young adults with disabilities aged 15 to 29 years. The findings of this review will provide insight into the impact of the COVID-19 pandemic on mental health, which may provide the basis for future research to develop effective interventions to manage such crises, as well as to inform government in the development of response plans for future pandemics which address the needs of young people with disabilities.

## Methods

### Search strategy

This systematic review follows the Preferred Reporting Items for Systematic Reviews and Meta-Analyses (PRISMA) guidelines [34] and was registered with the International Prospective Register of Systematic Reviews (CRD42022330204). A literature search was conducted including research published between March 2020 (when WHO declared the pandemic) and November 2022 using five electronic databases: Medline (Ovid), Embase (Ovid), PsycInfo (Ovid), Web of Science and CINAHL. A combined search of Medical Subject Headings (MeSH) and keyword terms were used for disability, COVID-19, mental health, and restricted to young people, aiming to identify all available studies (detailed Medline search strategy and history in Supplementary file 1). XYL conducted the search independently across five databases which was checked for accuracy by ZA.

### Inclusion criteria

Articles were considered eligible for inclusion if they met the following criteria: (1) peer-reviewed original articles published from March 2020 to November 2022; (2) quantitative studies; (3) study samples including individuals with disabilities (with no restriction on disability group or impairment type); (4) study samples restricted to adolescents and young adults aged 15–29 years, as mental health problems most likely to emerge in the mid-teens and mid-20s as people are in a transition to independence [35, 36] (defined as > 50% in the age range 15–29 years or results of the analysis disaggregated by age group); (5) included analyses comparing mental health outcomes before and after the COVID-19 pandemic or self-reported changes in mental health outcomes. There was no limitation for the country in which the study was conducted.

### Study selection

Retrieved data from databases were imported to Covidence online software [37] to eliminate duplicate articles and facilitate data screening. The studies were screened for eligibility by two independent reviewers (XYL, QTQ), first screening the titles and abstracts and secondly full text articles. All decisions were recorded on the Covidence platform and disagreements were resolved by a third reviewer (ZA).

### Data extraction

Data was extracted on the following characteristics: first author’s name, year of publication, location of the study, study design, ethics approval, informed consent, recruitment and sampling strategy, sample size, type of disability, exposure definition including comparison groups, data collection methods, time of data collection, mental health outcome definition, statistical methods, results. Data extraction was performed by one reviewer (XYL) and cross-checked by a second reviewer (ZA). Any discrepancies between them were resolved through discussion or consultation with other co-authors (MS, AK).

### Methodological quality assessment

The quality of included studies was evaluated using the Scottish Intercollegiate Guidelines Network (SIGN) tool for cohort studies [38]. The SIGN tool for cohort studies can be used to assess the quality of cohort studies in relation to 18 criteria within five broad categories, including measurement of exposures, measurement of outcomes, other forms of biases, the control of confounding, and statistical analysis. Each item in the checklist has answer options of “Yes”, “No”, “Can’t say”, “Does not apply”. An overall quality score for each paper is based on a grading criterion of: 1) low quality (0), where most of the items listed were not met (or not enough detail was provided to assess), or critical aspects of the study design were significantly flawed; 2) acceptable quality ( +), where most of the items listed were met, but there were some flaws in the study and associated risk of bias; 3) high quality (+ +), where a majority of the items listed were met with little or no risk of bias. Quality assessment was conducted by one reviewer (XYL) and discussed with three co-authors (ZA, MS, AK).

### Data synthesis

Due to the high heterogeneity of the data from the included studies and the small number of studies identified, a narrative synthesis was selected to synthesise the data. The data synthesis included summarising the results of each study, comparing the results between the studies for those with common mental health outcomes, and interpreting the results in light of the methodological quality assessments.

## Results

### Literature search

**Fig. 1:**
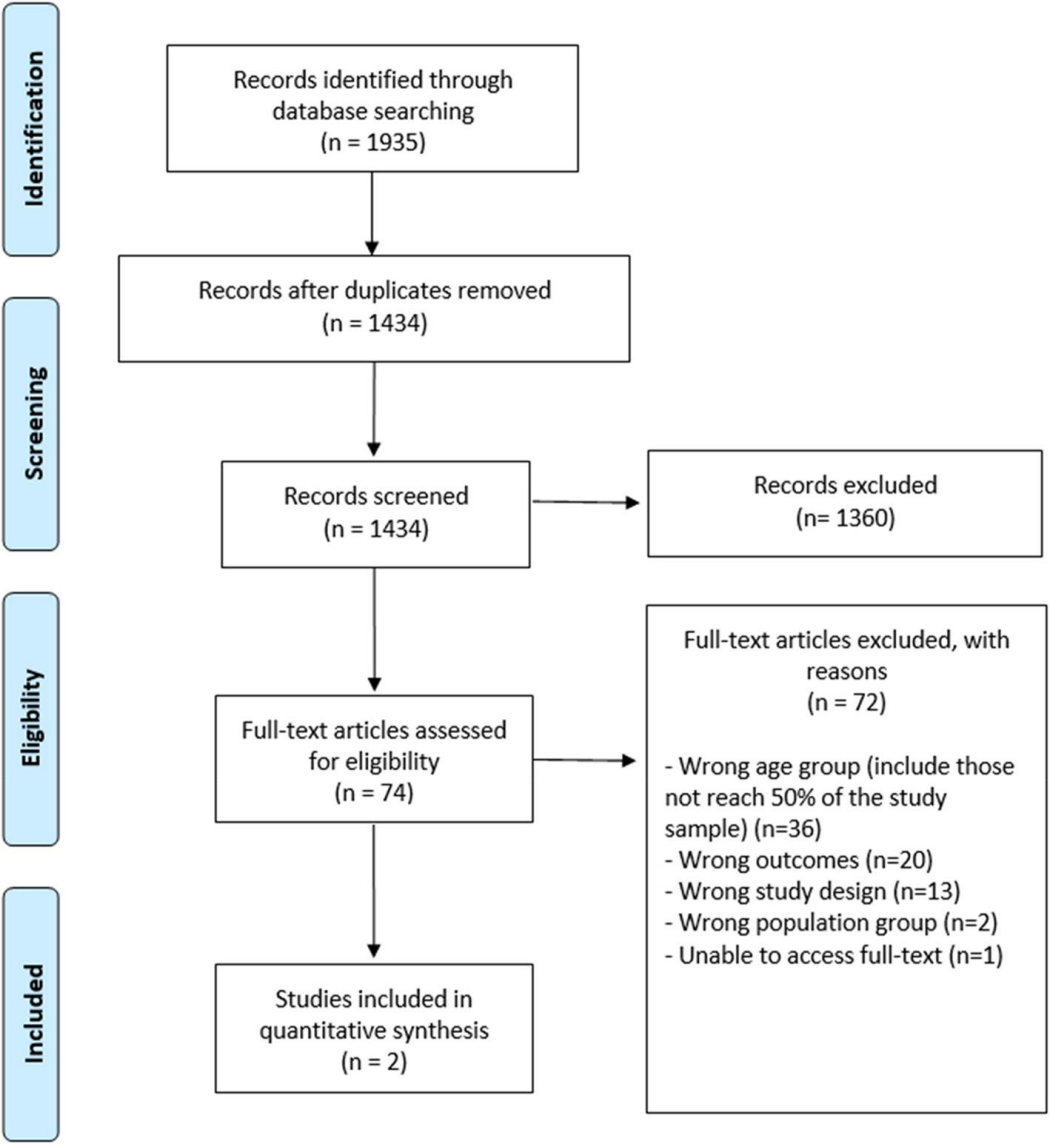
PRISMA flow diagram

The initial search yielded 1935 studies. After duplicates were removed, 1434 studies were screened for titles and abstracts, of which 76 full-text articles were assessed for eligibility. Of the 76 full-text articles screened, 2 studies met the eligibility criteria for inclusion in the systematic review and 74 were excluded. The PRISMA flow diagram of the study selection is illustrated in Fig. 1.

### Study characteristics

Study characteristics of the two included articles are summarised in Table 1. The total number of study participants across the studies was 532, with ages ranging from 18 to 35 years old. One study was a longitudinal study comparing a number of mental health outcomes (listed in Table 1) recorded at two different time points: at the start of the pandemic before the announcement of COVID-19 pandemic restrictions in the United States (20 March 2020) and during the pandemic while social distancing restrictions were in place (27 May 2020) [39]. At the second time point, an additional question was included in the survey which asked participants about the impact of COVID-19 on their emotional or mental health [39]. The other study was a cross-sectional study with a single time point where participants were asked retrospectively about changes in mental health problems or symptoms since before the pandemic [40].

**Table 1:** Characteristics of included studies

Authors. (country)	Study design	Timing	Sample size	Age	Disability	Mental health outcomes	Statistical methods	Main results
Adams et al., 2021 [39] (United States)	Longitudinal, online survey	Time 1: March 2020 Time 2: May 2020	*n* = 275	18–35 years	Autism spectrum disorder (ASD)	1) Depression, anxiety, general stress measured using the Depression Anxiety Stress Scale (DASS), measured at time 1 & 2. Changes in mental health symptoms for three subscales, assessed using 42 statements describing emotional symptoms, on a scale of 0 to 42 2) COVID-related distress, measured at time 2 using a single question: “To what extent has your emotional or mental health been negatively impacted by COVID-19?”	ANCOVA	1) Weak evidence of a difference in symptoms of depression, anxiety, and general distress between time 1 (pre-pandemic) and time 2 (during pandemic) 2) 58.1% reported COVID-19 had negatively impacted their emotional/mental health
Rosencrans et al., 2021 [40] (United States and Chile)	Cross-sectional, online and phone survey	Time 1: July 2020 (US) Time 2: October 2020 (Chile)	*n* = 208 (US) *n* = 49 (Chile)	18–30 years	Intellectual and development disabilities (IDD)	Change in mental health problems or symptoms, including a list of common mental health symptoms (being worried, stressed, scared, nervous, sad, angry, annoyed easily, impatient, tired, over-excited, jumpy, having problems sleeping, and other) measured at a single time point: “Are you having more mental health problems or symptoms?”	N/A	United States: 41% reported increased mental health problems. The mental health problems with the largest increases were worry (28.0%) and stress (27.2%) Chile: 56% reported increased mental health problems. The mental health problems with the largest increases were sadness (31.3%) and stress (28.1%)

Regarding the geographical location of included studies, one was conducted in the United States, [39] and one included a sample from each of the United States and Chile [40]. The disability population differed between the studies, with one focused on people with autism spectrum disorder (ASD), [39] and one on people with intellectual and developmental disabilities (IDD) [40]. The sampling strategy varied, with one study recruiting participants from an existing longitudinal study, [39] the other through emails, flyers, and social media [40]. Both studies conducted online surveys to collect data, and one of the studies also provided an alternative method of data collection, through telephone responses [40].

Although both studies collected information on common mental health outcomes, the mental health measures were different. One study used a validated mental health assessment instrument, the Depression Anxiety Stress Scale (DASS), which collects changes in emotional symptoms information for three subscales: the depression scale includes items that assess feelings of depression, hope and lack of interest in life; the anxiety scale includes items that measure somatic experiences related to anxiety and feeling anxious; and the general stress scale includes items that describe stress-induced emotions and actions [39]. These subscales were measured by asking participants to rate 42 statements describing emotional symptoms on a scale from 0 to 42 [39]. Furthermore, participants were asked about their responses to impacts of COVID-19-related distress defined as the extent to which their emotional or mental health was negatively impacted by COVID-19 in time 2. The impact responses were distributed into two categories (low impact or high impact) [39].

The second study used a self-developed survey, which adapted and modified the Epidemic-Pandemic Impacts Inventory (EPII), to be appropriate for use in the IDD population [40]. The study asked participants whether they were experiencing more mental health problems or symptoms during the COVID-19 pandemic to measure changes in mental health symptoms since before the pandemic, including a list of mental health problems (see Table 1) [40].

In the longitudinal study, ANCOVA tests were used to compare the mental health of people with ASD before and during the COVID-19 pandemic and percentages were presented to describe the proportion of the sample reporting, in the second wave, negative impacts of the pandemic and changes in mental health problems [39]. In the cross-sectional study, percentages were presented to describe the proportion of the sample experiencing more mental health problems or symptoms since before the pandemic, therefore no statistical test was conducted to assess the effect of the COVID-19 pandemic on mental health [40].

### Methodological quality of the studies and risk of bias

The quality of the two studies assessed using the SIGN cohort checklist is summarised in Table 2. The studies had clear research objectives, research questions, appropriate methods and outcomes, and reported informed consent of participants. However, the longitudinal study lacked reported information on data collection details, with details lacking on the online survey (questions and components), [39] and the other did not report granting of ethics approval for the study [40]. Regarding biases, for attrition bias, the longitudinal study reported a 13% drop out rate, which was within the acceptable rate (20%) according to the SIGN cohort checklist. Regarding assessment of the mental health outcome, while one outcome was measured prospectively in the longitudinal study, both studies included a retrospective question about the impact of the COVID-19 pandemic on their mental health, which may have introduced information bias [39, 40]. Neither study adjusted for potential confounders [39, 40]. Overall, both studies were rated as low quality [39, 40].

**Table 2:** Quality assessment of included studies according to the SIGN cohort checklist

**Item number**	**Description for cohort studies**	**Adams et al. **[39]** (2021)** **Longitudinal study**	**Rosencrans et al. **[40]** (2021)** **Cross-sectional study**
1.1	The study addresses an appropriate and clearly focused question. (Yes, No, Can't say)	Yes	Yes
1.2	The two groups being studied are selected from source populations that are comparable in all respects other than the factor under investigation. (Yes, No, Can't say, Does not apply)	Yes	No
1.3	The study indicates how many of the people asked to take part, did so in each of the groups being studied. (Yes, No, Does not apply)	Can’t say	Does not apply
1.4	The likelihood that some eligible subjects might have the outcome at the time of enrolment is assessed and taken into account in the analysis. (Yes, No, Can't say, Does not apply)	Can’t say	Does not apply
1.5	What percentage of individuals or clusters recruited into each arm of the study dropped out before the study was completed	13%	Does not apply
1.6	Comparison is made between full participants and those lost to follow up, by exposure status. (Yes, No, Can't say, Does not apply)	Does not apply	Does not apply
1.7	The outcomes are clearly defined. (Yes, No, Can't say)	Yes	Yes
1.8	The assessment of outcome is made blind to exposure status. If the study is retrospective this may not be applicable. (Yes, No, Can't say, Does not apply)	Does not apply	Does not apply
1.9	Where blinding was not possible, there is some recognition that knowledge of exposure status could have influenced the assessment of outcome. (Yes, No, Can't say)	Can’t say	Can’t say
1.10	The method of assessment of exposure is reliable. (Yes, No, Can't say)	Yes	Yes
1.11	Evidence from other sources is used to demonstrate that the method of outcome assessment is valid and reliable. (Yes, No, Can't say, Does not apply)	Yes	Yes
1.12	Exposure level or prognostic factor is assessed more than once. (Yes, No, Can't say, Does not apply)	Yes	Does not apply
1.13	The main potential confounders are identified and taken into account in the design and analysis. (Yes, No, Can't say)	No	No
1.14	Have confidence intervals been provided? (Yes, No)	Yes	No
2.1	How well was the study done to minimise the risk of bias or confounding? (High quality [+ +], Acceptable [ +], Low quality [0])	**0**	**0**
2.2	Taking into account clinical considerations, your evaluation of the methodology used, and the statistical power of the study, do you think there is clear evidence of an association between exposure and outcome? (Yes, No, Can't say)	No	No
2.3	Are the results of this study directly applicable to the patient group targeted in this guideline? (Yes, No)	No	No

### Study findings

In the longitudinal study of young people with ASD, Adams et al. found no statistically significant change in symptoms of depression, anxiety, and general distress from time 1 (pre-pandemic) and time 2 (during the pandemic) [39]. Despite this, 58.1% reported, in the second wave, that their emotional or mental health had been negatively impacted due to COVID-19 compared to pre-pandemic [39].

The cross-sectional study of young people with IDD found that 41.3% participants in the United States and 51.6% in Chile reported more mental health symptoms since the pandemic [40]. Regarding specific mental health symptoms, stress was the mental health condition which was most affected by the COVID-19 pandemic (United States: 27.2%; Chile: 28.1%), with more than a quarter of participants in both countries reported having more stress during the COVID-19 pandemic compared to pre-pandemic [40]. The study also reported increases in other mental health conditions including sadness (23.8% and 31.3%), worry (28.0% and 23.4%), problems sleeping (18.8% and 26.6%), being annoyed easily (19.3% and 23.4%), being impatient (17.8% and 26.6%), nervous (22.5% and 18.8%), angry (13.9% and 20.3%), scared (16.6% and 14.1%), tired (17.8% and 6.3%), jumpy (6.9% and 9.4%), over-excited (3.7% and 7.8%), and other (4.5% and 3.1%) [40]. The mental health problems with the largest increases since the COVID-19 pandemic were worry and stress in the United States and sadness and stress in Chile [40].

## Discussion

The objective of this review was to investigate the impact of the COVID-19 pandemic on the mental health of adolescents and young adults with disabilities. Although young people with disabilities account for one-third of the world's total disability population, we only identified two studies that met the inclusion criteria, with both assessed to be of low quality. This highlights the lack of research regarding the impact of the COVID-19 pandemic on the mental health of young people with disabilities and the urgent need for studies to investigate this research question.

Given that the pre-pandemic mental health of young people with disabilities is generally worse than their non-disabled peers, coupled with the life changes caused by COVID-19 restrictions, we had hypothesized that the pandemic would have had a significant impact on their mental health. Both studies found some evidence of a negative impact of the COVID-19 pandemic on the mental health of young people with disabilities [39, 40]. The cross-sectional study reported an increase in mental health symptoms for young people with IDD, particularly for stress. The longitudinal study, conducted in May 2020 in the first few months of the COVID-19 pandemic, found no evidence of a change in mental health symptoms for young people with ASD but participants reported negative impacts of the COVID-19 pandemic on their emotional or mental health. However, both studies were assessed to be of low quality and had small sample sizes, and there was a great deal of heterogeneity between the studies in terms of study population, geographical location, study designs, statistical approaches, and mental health outcomes.

The findings of the review suggest that the COVID-19 pandemic may have had negative impacts on the mental health of young people with disabilities. Perhaps even more importantly, the scarcity of studies and the poor quality of the available evidence underscore the uncertainty in the current evidence base addressing this important research question. This systematic review highlights that more relevant research is needed in the future to investigate the impact of the COVID-19 pandemic on mental health outcomes for young people with disabilities. Furthermore, both studies included in the systematic review were conducted early in the COVID-19 pandemic and only measured the short-term impact of the pandemic on mental health [39, 40]. Therefore, the medium- and long-term impact of the COVID-19 pandemic on the mental health of adolescents and young adults with disabilities is still unknown and needs to be explored by future research [39, 40]. Despite the marginalized position of adolescents with disabilities, none of the selected articles included people younger than 18 years and instead focused on young adults (aged ≥ 18 years), therefore adolescents with disabilities are a group which needs further attention. In addition, the studies included in the systematic review were restricted to young people with ASD and IDD. However, given the diversity of the experience of disability, the impact of the COVID-19 pandemic on the mental health of young people with other disabilities also needs to be explored. Considering that this research topic is relatively new, the above factors should be taken into account in future studies to further understand the impact of the COVID-19 pandemic on young people with disabilities. Though the increased availability of online services resulting from COVID-19 restrictions may have reduced some barriers to participation, the COVID-19 pandemic is likely to have exposed young people with disabilities to additional barriers in accessing health care, education, and employment. The impact of the COVID-19 pandemic on barriers to participation for young people with disability need to be understood, including whether these barriers may have led to poorer mental health outcomes.

To our knowledge, this is the first systematic review of published quantitative literature reporting impacts on the mental health of young people with disabilities before and after the onset of the COVID-19 pandemic. The systematic review was conducted in accordance with PRISMA guidelines, registered with PROSPERO, and the literature search was developed with guidance from expert librarians and experienced researchers. However, there are also limitations of the review. First, the review did not include grey literature, which may have contained research findings on the impact of the COVID-19 pandemic on the mental health of adolescents and young people with disabilities, which may have introduced publication bias. Second, the review only utilised 5 databases to conduct the search and identified a limited number of studies for inclusion. Third, the studies had high heterogeneity across the study characteristics, such as the study population, study design and the outcome measures, and both studies were assessed to be of low quality. As such, the findings of the review cannot be generalised to the broader population of adolescents and young adults with disabilities, and both studies were completed in 2020, precluding an understanding of medium and longer-term impacts of the pandemic on mental health outcomes.

In the event of future pandemics or other natural disasters, governments and institutions need to fully consider the needs of people with disabilities, including young people with disabilities, and tailor emergency preparedness plans to safeguard their well-being and to respond adequately to their needs. This review lays the foundation for future studies to investigate this important research question by addressing the limitations of the existing evidence. It highlights the urgent need to bridge the COVID-19 knowledge gap in this area by understanding the impact of the pandemic on the mental health of young people with disabilities.

## Conclusions

Though this systematic review provides some evidence that the COVID-19 pandemic may have had negative impacts on the mental health of young people with disabilities, it importantly highlights the lack of high-quality evidence and the urgent need for further research. Although high risk of bias and high heterogeneity are present in the studies included in this review, the findings of this review have the potential to lay the groundwork for improved studies in the future. Further research is essential to provide insights into the impact of the COVID-19 pandemic on the mental health of young people with disabilities. Understanding the relative differences between those with and without disabilities, differential effects between disability groups, as well as investigating the causal pathways leading to the detrimental mental health impacts, is important for governments and authorities to develop and promote pandemic planning and interventions tailored to the needs of the population of young people with disabilities.

## Supplementary Information

**Additional file 1.**

## Data Availability

All data generated or analysed during this study are included in this published article [and its supplementary information files].
