# Supplementary material for: A systematic review of the impact of the COVID-19 pandemic on the mental health of adolescents and young people with disabilities aged 15–29 years
Source: BMC Public Health. 2023 Jul 19;23:1390. doi: 10.1186/s12889-023-16260-z (PMC10357662; doi:10.1186/s12889-023-16260-z)
Supplement: Supplementary file 1 — Additional file 1. [file 12889_2023_16260_MOESM1_ESM.docx]

**Ancillary Material**

**Supplementary file 1.**

Search strategy for MEDLINE database.

| 1 | (disab* or handicap* or deaf or autism or autistic or asperger* or "down* syndrome" or "wheelchair user*" or "traumatic brain injur*" or "acquired brain injur*" or "spinal cord injur*").mp. |
| --- | --- |
| 2 | ((physical* or mobility or sensory or hearing or visual* or vision or development* or cognitive* or intellectual* or communication or language or speech or learning) adj2 (impaired or impairment* or disorder* or limitation* or loss or difficulty or difficulties)).mp. |
| 3 | disabled persons/ or persons with mental disability/ or amputees/ or visually impaired persons/ or persons with hearing impairments/ or exp autism spectrum disorder/ or intellectual disability/ or down syndrome/ or communication disorders/ or language disorders/ or learning disability/ or developmental disability/ or brain injuries, traumatic/ or spinal cord injuries/ or mobility limitations/ |
| 4 | 1 or 2 or 3 |
| 5 | (COVID or "COVID-19" or "COVID 19" or COVID19 or "SARS CoV 2" or "SARS CoV2" or SARSCoV2 or "SARSCoV 2" or "nCoV" or "novel CoV" or "coronavirus" or "corona virus" or "pandemic").mp. |
| 6 | COVID-19/ |
| 7 | (lockdown* or quarantin* or "physical distancing" or "social distancing" or "mask" or "masks").mp. |
| 8 | quarantine/ or physical distancing/ or masks/ |
| 9 | 5 or 6 or 7 or 8 |
| 10 | (psychological impact* or mental health or mental status or emotion* or mental* or cognit* or loneliness or depression or anxiety).mp. |
| 11 | depression/ or anxiety/ or mood/ or loneliness/ |
| 12 | 10 or 11 |
| 13 | (youth* or adolescent* or Adolescence or teen* or young adult* or Student* or young person).mp. |
| 14 | 4 and 9 and 12 and 13 |
